# Gamma variant vertically transmitted from a mild symptomatic pregnant woman associated with fatal neonatal COVID

**DOI:** 10.1016/j.bjid.2022.102385

**Published:** 2022-07-11

**Authors:** Walusa Assad Gonçalves-Ferri, Cristina Galdonyi Carvalheiro, Marisa Marcia Mussi-Pinhata, Bruna Pinto Dias Cavasin, Benedito Antonio Lopes da Fonseca

**Affiliations:** a Universidade de São Paulo (USP), Faculdade de Medicina de Ribeirão Preto (FMRP), Departamento de Pediatria, Ribeirão Preto, SP, Brasil; b Universidade de São Paulo (USP), Faculdade de Medicina de Ribeirão Preto (FMRP), Departamento de Medicina Interna, Ribeirão Preto, SP, Brasil

**Keywords:** Sars-CoV-2 variant, Vertical transmission, Covid 19, Preterm infant

## Abstract

Herein we describe a mild symptomatic real-time reverse transcriptase- polymerase chain reaction-confirmed coronavirus 2 (SARS-CoV-2) infection in a pregnant woman who gave birth to a preterm infant, 32 weeks gestational age. The neonate was immediately isolated after delivery and developed severe respiratory disease that progressed to multisystem inflammatory syndrome and death on the seventh day of life.

Genome sequencing detected the P.1 (gamma) variant in samples obtained at hospital admission (mother) and on the first (10h) and 13th days of life (neonate). Complete homology (mother's and newborn's sequences) confirmed vertical transmission.

To our knowledge, this is the first report of vertically-transmitted SARS-CoV-2 P.1 (gamma) variant in a mild symptomatic infection in pregnancy associated with fatal COVID in a neonate.

## Background

Severe acute respiratory syndrome coronavirus 2 (SARS-CoV-2) P.1 (gamma) is a variant that has circulated in Brazil. It was first detected in Amazonas state in late 2020^1^ and subsequently spread throughout Brazil. By May 2021, the coronavirus disease (COVID-19) pandemic in Brazil was at its worst, resulting in thousands of deaths.^2^ Available data on the P.1 (gamma) variant have suggested an increased transmissibility and a higher risk of reinfection than non-P.1 variants.^1^^,^^3^ However, its role in vertical transmission remains unknown.

Herein we report a case of SARS-CoV-2, P.1 (gamma) variant vertical transmission to a premature baby born to a mother with mild symptomatic COVID-19. The neonate developed severe respiratory disease that progressed to multisystem inflammatory syndrome (MIS-C) and death.

## Case report

A previously healthy 30-year-old pregnant woman (gravida 3, para 1, fetal loss 1) was admitted at Children Hospital of Ribeirão Preto Medical School in late March 2021 at 31 weeks and 4 days of gestation (calculated using ultrasound at 12 weeks) because of premature rupture of membranes. Considering her 3-day history of ageusia and anosmia, SARS-CoV-2 real-time reverse transcriptase polymerase chain reaction (RT-PCR) was performed on oral and nasopharyngeal swabs obtained at hospital admission, yielding positive results. The patient remained clinically stable without any respiratory symptoms. Her syphilis, HIV (human immunodeficiency virus), and toxoplasmosis serology screening in the first trimester and findings of vaginal-rectal cultures for Group B *Streptococcus* obtained two days before delivery were negative. Upon admission, her C-reactive protein (CRP) levels and complete blood count (CBC) were normal. She received two doses of betamethasone (6 mg/day) and enoxaparin (60 mg/day).

After remaining stable for four days, she developed fever (38^o^ C) with a shift to the left on CBC [6.1 × 10^3^/µL leukocytes (2% promyelocytes, 1% myelocytes, 4.2% bands)] and a high CRP level [7.2 mg/dL (normal value < 1.0)]. She was diagnosed with chorioamnionitis; thus, antibiotic treatment with clindamycin was initiated. At the onset of her spontaneous labor in breech presentation, cesarean delivery was performed in a negative pressure room under epidural anesthesia. The mother and staff wore correct personal protective equipment.

Delayed cord clamping, skin-to-skin contact, and breastfeeding were avoided, and contact between the neonate and mother after delivery was not initiated. The mother recovered her good clinical conditions, remained asymptomatic, and was discharged 3 days after delivery (Table 1).

**Table 1:** Timeline of clinical signs and symptoms, exams, and treatment of dyad.

Clinical data	Maternal evolution						Neonatal evolution			
	31 weeks	31 weeks and 4 days	31 weeks And 6 days	32 weeks and 1 day	32 weeks and 3 days	3 days after delivery	1-21 Days	22 -29 days	30-36 days	37-74 days
Diagnosis	-	Premature rupture of membranes	-	Chorioamnio nitis	Spontane ous labor in breech presentati on.	-	Neonatal respiratory distress syndrome	Septic shock	MIS-C	Death
Clinical data	Ageusia and anosmia	Ageusia and anosmia	Ageusia and anosmia	Fever (38C), Ageusia and anosmia	Ageusia and anosmia	Ageusia and anosmia	Seizures Respiratory distress Shock; Cardiac arrest (10 min)	Pulmonar y hypertension. (PAP: 60 mmHg) Shock.	MISC-C Heart: Biventricular concentric hypertrophy; Shock.	MIS-C Persistent hypoxemia and pulmonary hypertension Acute myocardial infarction Cardiac failure
Laboratoryfindings	-	Positive SARS-	Syphilis, HIV, and	Left shift on CBC			Ferritin: 821.90	Troponin e:1,175.00	Troponin e:15,780.10	Ferritin: 1515.6 NT-Pro-BNP (pg/mL)⁎
		CoV-2 RT-PCR	toxoplasmosis serology screening and vaginal- rectal cultures for Group B Streptococcus obtained were negative. C-reactive protein levels and complete blood count were normal.	[6.1 × 103/µL leukocytes (2% promyelocytes, 1% myelocytes, 4.2% bands)] and a high CRP level [7.2 mg/dL (normal value<1.0)]						>35,000
Treatme nt		Hospitaliza tion	Two doses of betamethasone (6 mg/day) and enoxaparin (60 mg/day).	Clindamycin was initiated	Cesarean delivery	Discharge (domiciliary isolation)	Exogenous surfactant; Mechanical ventilation; Vasoactive drugs; Enoxaparin; Azithromycin; Hydrocorti sone	iNO; Vasoactive drugs;	iNO; Vasoactive drugs	iNO; Sildenafil; Bosentan; Exogenous surfactant; Metroprolol; Intravenous immunoglobuli n; High-dose pulse steroids (methylprednisolone)

Abbreviations: iNO, inhaled nitric oxide; NT-pro-BNP, N-terminal pro B-type natriuretic peptide; PAP, pulmonary artery pressure.

⁎ Normal reference values: ferritin 22-322 ng/mL; NT-pro-BNP <125 pg/mL; PT 1.3 sec; troponin I <19 ng/dL.

The male infant weighed 2,160 g (adequate for gestational age) with 8/9 Apgar scores. He was immediately transferred to a separate room and did not need resuscitative measures. Non-invasive continuous positive airway pressure was initiated within 10 minutes of life due to mild respiratory distress. The neonate was transferred to a negative pressure isolation room in the neonatal intensive care unit (NICU). At two hours of life, chest radiography revealed diffuse bilateral interstitial pulmonary infiltrates. He was kept on nasal CPAP and received ampicillin and gentamicin for seven days for culture-negative, early-onset sepsis. Respiratory distress gradually resolved by the 4^th^ day of life (DOL), and the neonate was weaned from nasal CPAP. After being relatively stable with mild desaturations, on the 7^th^ DOL his condition rapidly deteriorated into critical respiratory and hemodynamic instability, complicated by a pneumothorax, cardiac arrest, and seizures (Table 1). Chest radiography showed massive bilateral coalescent opacities.

The neonate showed gradual clinical improvement, but on the 22^nd^ DOL, he was diagnosed with central line-associated septic shock caused by *Klebsiella oxytoca*, which resulted in severe pulmonary hypertension and hemodynamic instability. Despite treating the infection and negative control blood cultures at the 30^th^ DOL, his condition remained critical. Regarding his hemodynamic status, the newborn developed biventricular concentric cardiac hypertrophy and diastolic dysfunction, without coronary abnormalities on serial echocardiography. He was also diagnosed with myocardial infarction due to elevated cardiac injury biomarkers (Table 1) and electrocardiographic abnormalities. Additionally, the neonate had temperature instability (35.5-37.5^o^ C) from the 28^th^ to 35^th^ DOL, despite being in a temperature-controlled incubator or overhead radiant warmer.

During the septic episode, the neonate showed a worsening in inflammatory markers, which remained elevated even after the infection resolved (Table 1). Therefore, considering the history of COVID-19 prenatal exposure, presence of temperature instability, severe cardiovascular and respiratory compromise, and laboratory evidence of inflammation with no obvious bacterial infection, MIS-C diagnosis was considered. The neonate showed progressive worsening and died at 74 days of life.

### Virological testing results

SARS-CoV-2 RT-PCR results using nasal and oropharyngeal swabs obtained from the neonate at 10 hours of life and an endotracheal aspirate obtained on his 13th DOL were positive. Respiratory syncytial virus and influenza virus were not detected (endotracheal aspirate) on the 9th DOL.

To further evaluate SARS-CoV-2 vertical transmission, genome sequencing was performed on maternal and neonatal samples. The P.1 (gamma) variant of concern (20 J/501Y.V3) was sequenced for both samples. Genome analyses demonstrated 23 mutations in the sample sequences compared to those in the reference sequences, wherein 13 of them were located in the spike region. P.1 (gamma) defining mutations related to each genomic region were as follows: ORF1ab: S1188L, K1795Q; spike: L18F, T20N, P26S, D138Y, R190S, K417T, E484K, N501Y, H655Y, T1027I; Orf8: E92K and nucleocapsid: P80.

Additionally, we observed three other mutations in the spike: D614G, V1176F, and P681H. We also identified two mutations in the N protein (R203K, NG204R) and ORF1ab: ORF1b: P314L and ORF1b: E1264D. Complete homology between the mother's and newborn's sequences was observed. Sequencing of the neonate's endotracheal sample also showed complete homology with previously sequenced samples, indicating that he was suffering from active COVID-19.

### Laboratory techniques

Oral and nasopharyngeal swabs were collected upon the mother's hospital admission and neonate's first (10 hours) and 13^th^ DOL. SARS-CoV-2 RNA was detected from 100 µL of nasopharyngeal swab suspension. RNA extraction was performed using the Extracta Kit FAST DNA e RNA Viral (Loccus, SP, Brazil) in an automated extractor (EXTRACTA 32; Loccus) following the manufacturer's guidelines. SARS-CoV-2-RT-PCRs were performed using the Gene FinderTM COVID19 Plus RealAmp kit (OSang Healthcare Co. Ltd.), which detects RdRp, E, and N genes. The reaction protocol was performed according to the manufacturer's protocol using the 7500 Real-Time PCR System (Thermo Fisher Scientific).

### Sequencing

SARS-CoV-2 complete genomic sequences were obtained through Illumina COVIDSeq technology according to the manufacturer's protocol. Sequencing libraries were pooled, normalized to 4 nM, and denatured with 0.2 N NaOH and 400 mM Tris-HCl (pH-8). Each sample library (9 pM) was loaded onto a 300-cycle MiSeq Nano Reagent Kit v2 and run on an Illumina MiSeq instrument (Illumina, San Diego, CA, USA).

### Bioinformatic analysis

Raw sequence data were subjected to quality control analysis using FastaQC^4^ software version 0.11.8. Trimming was performed using Trimmomatic version 0.3.9^5^ to select best quality sequences. Bioinformatics analyses were performed on sequences with quality scores > 30. We mapped the trimmed sequences against the SARS-CoV-2 reference (GenBank refseq NC_045512.2) using BWA (Burrows-Wheeler Aligner)^6^ software and samtools^7^ for read indexing. Mapped files were submitted to refinement with the Pilon^8^ software to obtain the most accurate information on indels and insertions. Afterwards, the trimmed sequences were subjected to a remap against the genome refined by Pilon. Finally, we used bcftools^9^ for variant calling and seqtk^10^ to create a consensus genome.

### Phylogenetic analysis

A representative subset of 3,874 genomes obtained from GISAID was obtained following the Nextstrain guidelines. Two full-length novel genomes were appended to this subset for further analysis. Sequence alignment was performed using MAFFT^5^ v7.475 and manually curated to remove artifacts using Aliview.^11^ Maximum likelihood (ML) phylogenetic trees were estimated using IQtree^12^ v.16.12, applying the ML algorithm with statistical support of ultrafast bootstrap with 1000 replicates. The nucleotide substitution model was GTR+G4+F, chosen according to the Bayesian information criterion statistical model. The final formatting and visualization of the phylogenetic tree were performed using the ggtree R package.^13^

### Mutational pattern analysis

Mutational profiles were investigated using the Nextclade tool to describe substitutions. Subsequently, the set of non-synonymous mutations was compared to the profiles available in the PANGO lineage resource to attribute genomes to lineages.

### Sequencing information

The mother's and newborn's samples yielded 188,205 reads with a mean depth of 675,948 and 99.93% coverage and 154,023 reads with a mean depth of 593.93% and 99.92% coverage, respectively.

### Ethics approval

The study was approved by the Research Ethics Committee of the Medicine School in Ribeirão Preto, University of São Paulo, Brazil (CAAE: 48798421.7.0000.5440 - 4.835.538/2021) and from the Brazilian hospitals and maternal services. The parents agreed and signed the consent to data publishing.

## Discussion

To our knowledge, this is the first documented case of SARS-CoV-2 P.1 (gamma) variant vertical transmission, from mild symptomatic mother, associated with fatal COVID-19 in the neonate, starting with respiratory failure complicated by MIS-C and evolving to neonatal death.

Intrauterine fetal exposure to SARS-CoV-2 was confirmed by a positive RT-PCR from the neonate's sample collected 10 hours after birth, suggesting mother-to-child transmission.^14^ This finding was not associated with direct contact between the mother and infant as they were separated immediately after delivery.^3^^,^^14^, ^15^, ^16^, ^17^ Most important for the evaluation of SARS-CoV-2 vertical transmission is the finding of a complete homology between the mother's and newborn's sequences, evidencing their origin from a single source. Furthermore, the detection of SARS-CoV-2 on a sterile sample obtained from the neonate on the 13^th^ DOL confirmed his ongoing infection.

Effects of SARS-CoV-2 infection in fetuses and newborn infants remain unknown. Recent systematic reviews on neonates born to SARS-CoV-2-infected mothers reported vertical transmission rates of 3.2%-4.2%, based on positive RT-PCRs using nasopharyngeal swabs obtained before 48 hours of life. However, RT-PCR, which is the gold standard diagnostic test, was not universally performed and was mostly obtained after 48 hours of life.^3^^,^^15^, ^16^, ^17^, ^18^ Thus, the description of vertical transmission in the literature is frequently incomplete, hindering confirmation of this mode of transmission. Also, the report of vertical transmission in a mild COVID pregnant woman is scarce, leading to sub notification of mother-to-child SARs-CoV-2 transmission.

Tropism of SARS-CoV-2 to the fetus is a concern, as the angiotensin-converting enzyme 2 receptor used by the virus to invade cells, is found in placental cells and fetal tissues.^17^ Most reports of neonatal COVID-19 have notably described the presence of mild symptoms, with approximately 2% of newborns requiring NICU admission.^18^ The most commonly reported signs and symptoms were respiratory abnormalities (52.5%), fever (44.3%), and gastrointestinal (36%), neurological (18.6%), and hemodynamic manifestations (10.3%).^18^ However, MIS-C is a rare manifestation of SARS-CoV-2 in children, more common in older children, and neonates seem less affected.^19^ Only a few cases of MIS-C in newborns have been reported, although none had a definite association with vertical transmission.^15^^,^^18^^,^^20^

Since 2020, few case reports of SARs-CoV-2 vertical transmission have been described; in Table 2, we presented the case reports of newborns from mothers positive for coronavirus and her infants who presented SARS-CoV-2 RT-PCR positive, indicating vertical transmission. We only considered infants in whom nasal RT-PCR was positive without previous contact with the mother. We showed seven cases, and only one identified the variant (Delta variant – B1) associated with the vertical transmission.^21^, ^22^, ^23^, ^24^, ^25^, ^26^, ^27^

**Table 2:** Case reports characteristics of infants with a diagnosis of SARS-CoV-2 vertical transmission by RT-PCR.

Author/Year	Country	HOL (h)	GA (weeks)	Clinical signs	Outcome	Variant
Zamaniyan et al.^21^ 2020	Iran	1	32	Fever	Not discharge at the publication time, but well	-
Kulkarni et al.^22^ 2021	India	38	38,2	Fever, poor feeding, and hyperbilirubenemia	Discharge at 21 DOL	-
Verheijen et al.^23^ 2022	Netherlands	19	36,7	Respitarory distress; MV for 22 days	Discharge/resolution	B.1.177
Alzamora et al.^24^ 2022	Peru	16		Respiratory distress	Discharge/ resolution	-
Morales,^25^ 2022	Spain		31,6	Respiratory distress, 12h of MV, tachycardia and myocarditis	Discharge/resolution	-
Kalani-moghaddam et al.^26^ 2022	Iran	1	39,4	Respiratory distress, MV for 4 days, hemodynamic instability	Discharge at 20 DOL	-
Malek et al.^27^ 2022	Indian	2	27	Respiratory distress (CPAP), MISC	Discharge at 24 DOL	-
Sanches et al.^28^ 2022	USA		30	Pneumonia, MV for 12 days	Death at 12 DOL	-
Gonçalves-Ferri et al. 2022	Brazil	7	32	Respiratory distress, MISC, MV for 74 days	Death at 74 DOL	P.1

GA, gestational age; HOL, Hours of life; DOL, Days of life; MV, Mechanical ventilation; MISC, Multisystem inflammatory syndrome.

This is the first case of SARS-CoV-2 P.1 (gamma) variant vertical transmission in a mild symptomatic mother, with confirmed mother-to-child transmission, followed by probably neonatal MIS-C and death. Considering that an association between the severity of neonatal disease and maternal P.1 (gamma) variant infection cannot be ruled out, this report raises concerns about the impact of infection by this specific variant on maternal - neonatal health.

## Conclusion

SARS-CoV-2 vertical transmission is a possible finding and can be associated with fatal neonatal COVID, even in neonates born to a mild symptomatic woman. In the face of the different variants that have emerged worldwide, further studies focusing on infection by different variants and neonatal outcomes are essential to understand better the repercussions of infection by distinct SARS-CoV-2 variants in pregnant women and their newborns.

## Conflicts of interest

The authors declare no conflicts of interest.
